# Influences of organic nitrogen application ratio on oil content in flue-cured tobacco based on field experiments and a random forest model

**DOI:** 10.3389/fpls.2026.1767538

**Published:** 2026-03-02

**Authors:** Zijun Sun, Wanhui Jiang, Huaiyuan Li, Yaoxing Liang, Xin Yang, Chen Peng, Lanjun Shao, Qihang Yang, Jijiao Fu, Jianjun Chen, Shiyuan Deng

**Affiliations:** 1 College of Agriculture, South China Agricultural University, Guangzhou, China; 2 Center for Basic Experiments and Practical Training, South China Agricultural University, Guangzhou, China; 3 China Tobacco Guangdong Industrial Co., Ltd., Guangzhou, China

**Keywords:** flue-cured tobacco, glandular trichome development, lipid metabolism, oil content, organic nitrogen ratio, random forest model

## Abstract

Tobacco is a key economic crop, with leaf oil content serving as a critical determinant of leaf quality. To address the limited understanding of mechanisms underlying oil content improvement and the decline in flue-cured tobacco quality caused by long-term reliance on chemical fertilizers, this study integrated field experiments with a machine learning approach. Five treatments with varying organic nitrogen ratios (0%, 10%, 20%, 30%, and 40%) were evaluated at a single experimental site in Hengyang, Hunan. Results indicated that a 30% organic nitrogen ratio significantly enhanced the activity of key lipid metabolism enzymes, promoted the accumulation of lipid metabolites (including cembratriene-diol and sucrose esters), increased glandular trichome density, and improved leaf physical properties such as softness, tensile strength, and thickness, ultimately achieving the highest oil content. Using a robust data augmentation strategy and Recursive Feature Elimination, a Random Forest model was constructed to dissect the complex regulatory network. The model achieved a high predictive accuracy (CV R² = 0.819) on the augmented dataset, significantly outperforming the model based on original small-sample data. Feature importance analysis identified petroleum ether extract, cembratriene-diol, leaf softness, reducing sugar, and glandular trichome density as the primary predictors. Significant interactions among these features were also revealed by SHAP dependence plots. These findings provide a theoretical basis for optimizing organic nitrogen application to enhance tobacco leaf oil content and quality in agricultural production.

## 1 Introduction

Flue-cured tobacco is one of the most important economic crops, with China’s cultivation area ranking first worldwide (Poltronieri, 2006; Chen et al., 2019). Fertilization plays a pivotal role in determining yield and quality (Bender et al., 2023), yet excessive or imbalanced application can degrade soil resources, waste inputs, and disrupt leaf chemistry (Yan et al., 2025). The long-term, sole use of chemical fertilizers has become widespread in flue-cured tobacco production, but it reduces soil fertility and deteriorates soil physical and chemical properties, undermining the conditions necessary for producing high-quality tobacco leaves (Gruber and Galloway, 2008).

Organic fertilizers, sourced from organic waste, supply abundant organic matter and micronutrients while improving soil structure, water retention, fertility, and aeration (Lu et al., 2024; Shang et al., 2020). However, their sole application is constrained by slow nutrient release and higher costs (Wang et al., 2020). Consequently, combining organic and inorganic fertilizers has emerged as a key strategy in sustainable flue-cured tobacco cultivation (Paramesh et al., 2023).

Nitrogen, an essential macronutrient, is frequently the most limiting factor for plant growth and yield (Zhou et al., 2023). Overuse of inorganic nitrogen not only wastes resources but also contributes to environmental degradation (Shi et al., 2023). Optimizing the ratio of organic to inorganic nitrogen can mitigate these issues, improving yield and quality while balancing internal chemical composition (Dinesh et al., 2024; Yang et al., 2023). Appropriate organic nitrogen supplementation can promote protein synthesis and enhance oil accumulation, directly contributing to the desirable oiliness of the final cured leaves (Qiu et al., 2020; Zhang et al., 2021).

In flue-cured tobacco grading, “oil content” (sensory score) is a composite, sensory-driven metric reflecting the leaf’s surface appearance, texture, and aroma (Gao et al., 2025). It is subjectively evaluated by experts based on visual and tactile cues. In contrast, Petroleum Ether Extract (PEE) represents the chemical fraction of lipids and other non-polar compounds extracted from the leaf, serving as a quantitative proxy for total lipid content. These characteristics are intricately linked to a network of physical and chemical properties. For instance, increased glandular trichome density elevates the levels of cembratriene-diol (CBT-diol) and sucrose esters (Philippe et al., 2022; Qu et al., 2018). As primary constituents of PEE, these metabolites constitute the material basis for the leaf’s oily appearance and aroma, thereby influencing the sensory evaluation. Biochemically, the accumulation of these lipid compounds is governed by key enzymes that dictate metabolic flux. Fatty acid synthase (FAS) is central to *de novo* fatty acid synthesis, directly promoting fatty acid accumulation (Chen et al., 2025). Phosphoenolpyruvate carboxylase (PEPC), a key enzyme in carbon fixation, supplies precursors for amino acid and organic acid synthesis, thereby influencing overall metabolic flux and strengthening carbon-nitrogen synergy, which in turn promotes the accumulation of total and reducing sugars (Fan et al., 2013). Furthermore, phosphatidic acid phosphatase (PAP) is critical for lipid metabolism and phospholipid regulation, with its catalyzed reactions being crucial steps for triacylglycerol synthesis and storage (Carman and Han, 2009, Carman and Han, 2006). The collective activity of these enzymes influences the levels of pyruvic acid (PA), total phenols (TP), and other sugars, indirectly shaping the final lipid composition and sensory profile of cured leaves. However, the strong interdependence of these structural and biochemical indicators often causes severe multicollinearity, complicating efforts to isolate each factor’s true contribution using conventional statistical approaches.

To address these challenges and elucidate the underlying mechanisms, this study employs a Random Forest (RF) model, which effectively captures non-linear relationships, variable interactions, and high-dimensional dependencies while remaining robust to noise and overfitting (Swamy et al., 2025). As a non-parametric method, RF builds an ensemble of Classification and Regression Trees (CART) (Seifert et al., 2020). These capabilities have led to its wide adoption in diverse disciplines, such as ecological prediction and expression-based interpretation (Xavier and Wilfried, 2009; Bureau et al., 2005). RF is a powerful ensemble learning method capable of handling non-linear relationships and high-dimensional interactions (He et al., 2024), making it ideal for dissecting complex agronomic traits. To enhance interpretability, we combined RF with feature attribution and visualization tools, including Mean Decrease in Impurity (MDI) for feature importance, Partial Dependence Plots (PDP), and SHAP (SHapley Additive exPlanations) value analysis. This multifaceted approach aims to yield a transparent and interpretable model (Bouni et al., 2024; Bénard et al., 2022), enabling both global and local insights into how key physicochemical indicators regulate oil content and reveals complex interactions among them.

This study aims to (1) elucidate the mechanisms by which organic nitrogen ratios influence the oil content of flue-cured tobacco through key physicochemical pathways, (2) identify the optimal organic nitrogen ratio for maximizing oil content, and (3) establish a data-driven framework for more precise evaluation and management of tobacco leaf quality in agricultural production. Here, we show that a 30% organic nitrogen ratio achieves the highest oil content by optimizing a suite of interconnected physicochemical traits. Using an optimized RF model on augmented data, we identified PEE, CBT-diol, and leaf softness as the most influential predictors. Our results further reveal that this optimal nitrogen ratio promotes oil content by stimulating lipid metabolism and glandular trichome development, establishing a clear, data-driven connection between fertilization strategy and final leaf quality.

## 2 Materials and methods

### 2.1 Experimental materials and site description

The field experiment was conducted from March to July 2024 in Hexi Village, Guanshi Town, Hengnan County, Hengyang City, Hunan Province, China (26°43′12″N, 112°50′55″E; altitude 70 m), The region has a subtropical monsoon climate, with an average temperature of approximately 24 °C and precipitation of 800 mm during the tobacco growing season (March-July). The flue-cured tobacco variety used in this study was ‘Yunyan 87’. The site, previously cultivated with rice, had the following basic soil properties: pH 7.46, total nitrogen 2.47 g·kg^-^¹, total potassium 1.69 g·kg^-^¹, total phosphorus 0.95 g·kg^-^¹, alkali-hydrolyzable nitrogen 90.44 mg·kg^-^¹, available potassium 379.90 mg·kg^-^¹, available phosphorus 49.63 mg·kg^-^¹, and organic matter 43.16 g·kg^-^¹.

### 2.2 Experimental design

The experiment was arranged in a randomized complete block design with three replicates. Under a constant total N application rate (165 kg·N·ha^-^¹), five treatments with varying proportions of organic N were established: ON0 (0% organic N), ON10 (10%), ON20 (20%), ON30 (30%), and ON40 (40%), totaling 15 plots. Guard rows were established around the experimental area, and the planting density was maintained at 17,250 plants ha^-^¹. The fertilizers used included a specialized basal fertilizer for tobacco (8% N, 10% P_2_O_5_, 11% K_2_O), a starter fertilizer (20% N, 9% P_2_O_5_), a specialized topdressing fertilizer for tobacco (10% N, 32% K_2_O), potassium sulfate (52% K_2_O), potassium magnesium sulfate (25% K_2_O), and superphosphate (12% P_2_O_5_). The organic N source was a bio-fermented rapeseed cake fertilizer (2.4% TN, 1.05% TP, 2.55% TK). All other agronomic practices followed the 2024 technical guidelines provided by the Hengyang City Company of the Hunan Provincial Tobacco Monopoly Bureau. Detailed fertilization data for each treatment are provided in **Supplementary Table 1**.

### 2.3 Sample collection and classification

To ensure consistency, samples were collected at two distinct stages targeting specific physiological and quality indicators. First, 90 days after transplantation, fresh leaf samples were collected from the sixth leaf position (starting from the top), and 10 leaves were collected from each replicate. After removal of the veins, the leaves were stored at -80°C for determination of physiological and biochemical indexes, including enzyme activities (FAS, PEPC, PAP) and metabolite contents (PA, TP). Second, after the flue-curing process, cured leaves of the B2F grade (upper leaves) were selected to measure chemical components (PEE, TS, RS), physical properties (softness, tensile strength, thickness), and the sensory oil content score.

### 2.4 Determination of lipid metabolism-related products

The contents of pyruvic acid (PA), total phenols (TP), and the activities of FAS, PEPC, and PAP were measured using reagent kits from Suzhou Grace Biotechnology Co., Ltd. (Suzhou, China). The mass of all measured samples was 0.1 g, and the volume of solvent was 1 mL.

The content of PA was determined based on its reaction with 2,4-dinitrophenylhydrazine to form 2,4-dinitrophenylhydrazone, a brownish-red hydrazone in an alkaline solution. Absorbance was measured at 520 nm.

The TP content was measured using the Folin-Ciocalteu method, based on phenol-induced reduction of tungstomolybdic acid, producing a blue complex (760 nm).

FAS activity was monitoring NADPH consumption (absorbance at 340 nm) during conversion of acetyl-CoA and malonyl-CoA to long-chain fatty acids.

PEPC activity was assessed through coupled reactions with malate dehydrogenase, tracking NADH oxidation at 340 nm. PAP activity was determined via β-glycerophosphate hydrolysis, detecting liberated inorganic phosphate using a phosphorus-specific reagent.

All assays were conducted in 96-well microtiter plates, with triplicate technical replicates.

The contents of total sugar (TS) and reducing sugar (RS) were determined following YC/T 159-2002, and petroleum ether extract (PEE) was measured according to YC/T 176-2003.

### 2.5 Observation and density statistics of glandular trichomes

During the germination period, the upper surface of the first mature leaf (from top to bottom) was taken, stained with 0.2% (w/v) Rhodamine B for 30 minutes, rinsed three times with distilled water, and blotted dry. Glandular trichome density was quantified using a digital microscope (VHX-500F, KEYENCE Corporation, Japan) by analyzing three random fields from the central upper epidermis.

### 2.6 Determination of glandular trichome secretions

The first mature leaf (from top to bottom) was taken during the germination period. Leaf discs with a diameter of 5 cm were cut from the leaves. Fifty leaf discs constituted one biological replicate, and three biological replicates were prepared for each material. The leaf discs were extracted by dipping them eight times (2 s each) into a dichloromethane solution. A 1 mL internal standard solution (a mixture of 2.020 mg·ml^-^¹ sucrose octaacetate and 2.542 mg·ml^-^¹ n-heptadecanol) was added to the extract. After thorough mixing, the solution was filtered and then concentrated using a rotary evaporator (LCA-RN-1300BE, Shanghai Lichen Instrument Technology Co., Ltd.). The concentrate was dried under a nitrogen stream using a nitrogen evaporator (LC-DCY-12GP, Shanghai Lichen Instrument Technology Co., Ltd.). The dried residue was subsequently silylated and analyzed by GC-MS (7890B-5977A, Agilent Technologies Inc., USA).

### 2.7 Measurement of physical properties of cured leaves

The leaf softness value (mN) was measured longitudinally and transversely using a softness tester (YT-RRY1000, Hangzhou Yante Technology Co., Ltd.). The tensile strength was determined with a force gauge (SF-10, Wenzhou Weidu Electronics Co., Ltd.), and the thickness was determined using a high-precision thickness gauge (547-401, Mitutoyo Corporation, Japan).

### 2.8 Evaluation of oil content in cured tobacco leaves

Oil content was scored based on GB 2635-92 (“Grading of Flue-cured Tobacco”), supplemented by expert input: abundant (9–10 points), present (6–8 points), slightly present (3–5 points), and sparse (0–2 points). Evaluators were blinded to the specific treatments to minimize bias. The scores were treated as a continuous variable for regression modeling, a common practice in sensory analysis to capture subtle quality gradients.

### 2.9 RF model construction and design

The model was developed using the RandomForestRegressor class from the scikit-learn library in Python. The standardized key indicators were used as input features, and the original oil content scores served as the target variable. To overcome the limitations of a small sample size (N = 15 biological replicates) and to capture robust non-linear relationships, we implemented a comprehensive modeling framework involving data augmentation, recursive feature elimination (RFE), and hyperparameter optimization.

#### 2.9.1 Data augmentation strategy

We employed a two-step data augmentation strategy to expand the dataset to N = 45, thereby enhancing model generalizability. First, synthetic samples were generated by injecting Gaussian noise into the original feature vectors, scaled to 3% of the standard deviation of each feature to simulate natural biological variability. Second, linear interpolation was performed between randomly selected pairs of original samples, mimicking intermediate physiological states. A random seed was fixed to ensure reproducibility.

#### 2.9.2 Feature selection via recursive feature elimination

To address potential multicollinearity and identify the most informative predictors, we utilized Recursive Feature Elimination with Cross-Validation (RFECV). This method iteratively removed the least important features based on model accuracy until the optimal feature subset was identified. The selection process was guided by maximizing the R² score under 3-fold cross-validation, ensuring that the final model relied only on non-redundant, high-impact indicators.

#### 2.9.3 Model optimization and training

The Random Forest model was trained on the augmented dataset using the selected features. Instead of arbitrary parameter setting, we employed GridSearchCV to systematically optimize key hyperparameters, including the number of estimators (n_estimators), maximum tree depth (max_depth), and minimum samples per leaf (min_samples_leaf). The final model’s prediction for a new sample $x^{\prime }\;$ is the average of the predictions from all B decision trees in the forest. The equation is shown as Equation 1.

(1) $$\begin{array}{c} \hat{f_{rf}}\left(x^{\prime }\right)=\frac{1}{B}\sum_{b=1}^{B}T_{b}\left(x^{\prime }\right) \end{array}$$

where B is the total number of decision trees in the forest, and $T_{b}\left(x^{\prime }\right)$ represents the prediction of the b-th tree for sample $x^{\prime }$.

#### 2.9.4 Model evaluation and interpretation

The overall performance of the model was evaluated using both Out-of-Bag (OOB) estimation and K-fold Cross-Validation (CV, K = 3). The OOB R² provides an estimate of the model’s generalization performance by using the one-third of the data left out of the bootstrap sample for each tree as a validation set. The cross-validation R² is calculated by partitioning the data into K mutually exclusive subsets, iteratively training the model on K-1 subsets and testing on the remaining one, and then averaging the performance. The equation is shown as Equation 2.

(2) $$\begin{array}{c} R_{CV}^{2}=1-\frac{\sum_{i\in \text{TestData}}{\left(y_{i}-\hat{y_{i,CV}}\right)}^{2}}{\sum_{i\in \text{TestData}}{\left(y_{i}-\bar{y}\right)}^{2}} \end{array}$$

where $\hat{y_{i,CV}}$ is the prediction for sample i when it was in the held-out fold, $y_{i}$ is the true value, and $\bar{y}$ is the mean of the true values for all samples.

To gain deeper insights into the RF model and uncover underlying mechanisms, we first quantified the global relative importance of each input feature in predicting oil content by calculating the Mean Decrease in Impurity (MDI). Second, we utilized Partial Dependence Plots (PDP) to visualize the marginal effect of changing a single feature on the model’s average prediction. The equation is shown as Equation 3.

(3) $$\begin{array}{c} \hat{f_{j}}\left(x_{j}\right)\approx \frac{1}{n}\sum_{i=1}^{n}\hat{f}\left(x_{j},x_{C}^{\left(i\right)}\right) \end{array}$$

where $\hat{f}$ is the trained RF model, n is the number of samples, and $x_{C}^{\left(i\right)}$ represents the values except feature j for the i-th sample.

Concurrently, SHapley Additive exPlanations (SHAP) value analysis was introduced. Using the TreeExplainer algorithm from the shap library, we calculated the contribution of each feature to the final prediction for every individual sample. SHAP values satisfy the property of additivity, meaning the difference between an individual prediction and the average prediction is equal to the sum of the SHAP values for all features of that sample. The equation is shown as Equation 4.

(4) $$\begin{array}{c} \hat{f_{rf}}\left(x_{i}^{'}\right)-E\left[\hat{f_{rf}}\left(X^{\prime }\right)\right]=\sum_{j=1}^{D}\phi_{ij} \end{array}$$

where $\phi_{ij}$ is the contribution of feature j to the prediction for sample i, and D is the feature dimension.

These values were visualized through SHAP summary and dependence plots to provide a more comprehensive local and global model interpretation. Model diagnostics were performed by analyzing the distribution of cross-validation residuals (Shapiro-Wilk test for normality), the relationship between residuals and predicted values, and Q-Q plots.

### 2.10 Data processing and statistical analysis

Data were processed using Excel 2021, SPSS 19.0, and Pycharm 2024. Line graphs and bar charts were produced with OriginPro 2024. One-way analysis of variance (ANOVA) followed by Duncan’s new multiple range test was used for variance analysis. The Spearman method was used to calculate correlation coefficients.

## 3 Results

### 3.1 Glandular trichome density and secretion content

**Figure 1:**
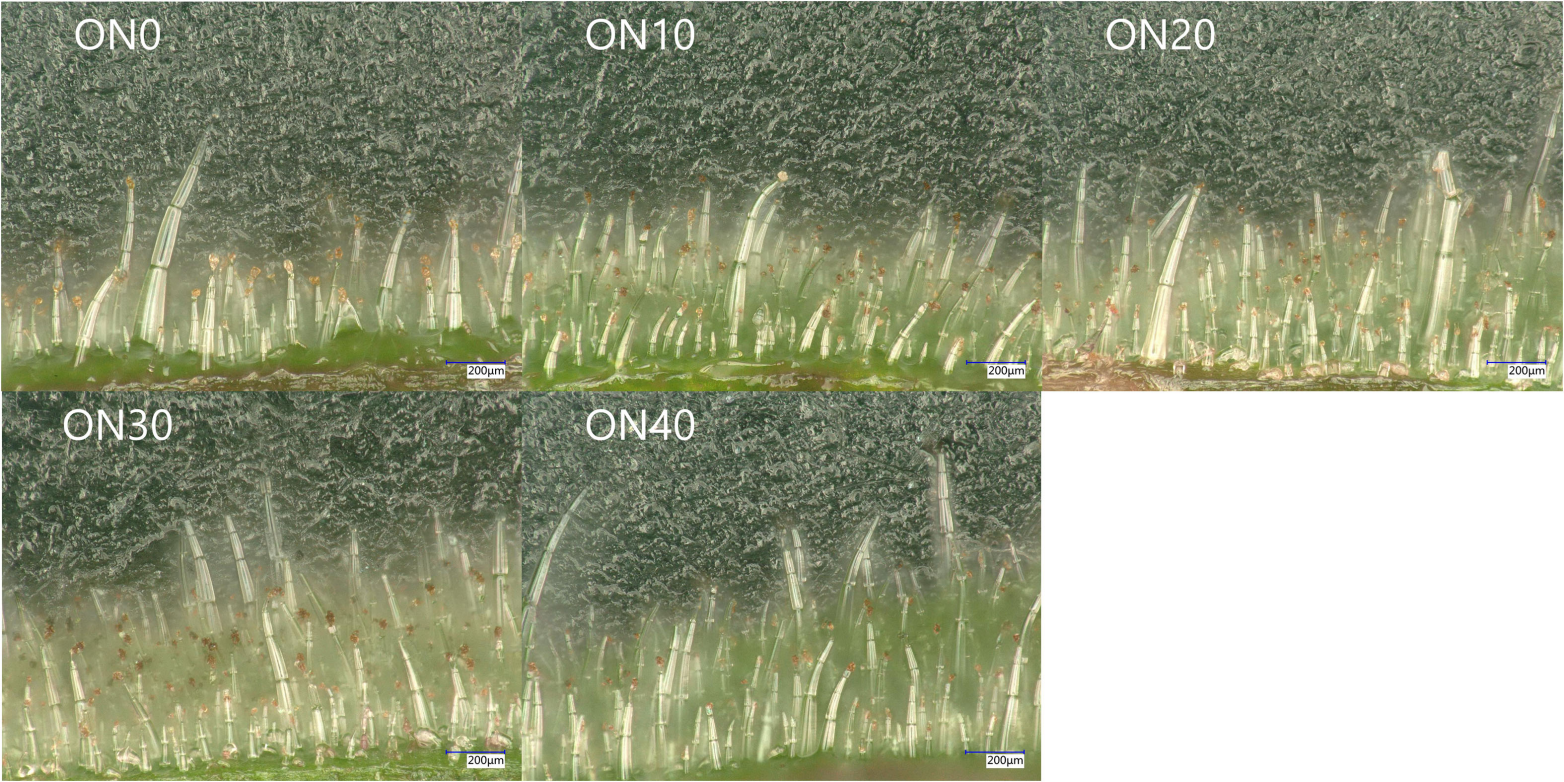
Morphology of glandular trichomes on flue-cured tobacco leaves under different organic nitrogen treatments. Representative Ultra-Depth Three-Dimensional microscope images showing the cross-sections of fresh tobacco leaf epidermes at the budding stage. The panels display the effects of treatments with varying organic nitrogen to total nitrogen ratios: ON0 (0%), ON10 (10%), ON20 (20%), ON30 (30%), and ON40 (40%). The images show various trichome types, including prominent long-stalked glandular trichomes (characterized by multi-cellular stalks and secretory heads) and shorter-stalked glandular trichomes. An increase in trichome density, particularly of the long-stalked type, is observable in the ON30 treatment compared to the ON0 control. Scale bars = 200 μm.

**Figure 2:**
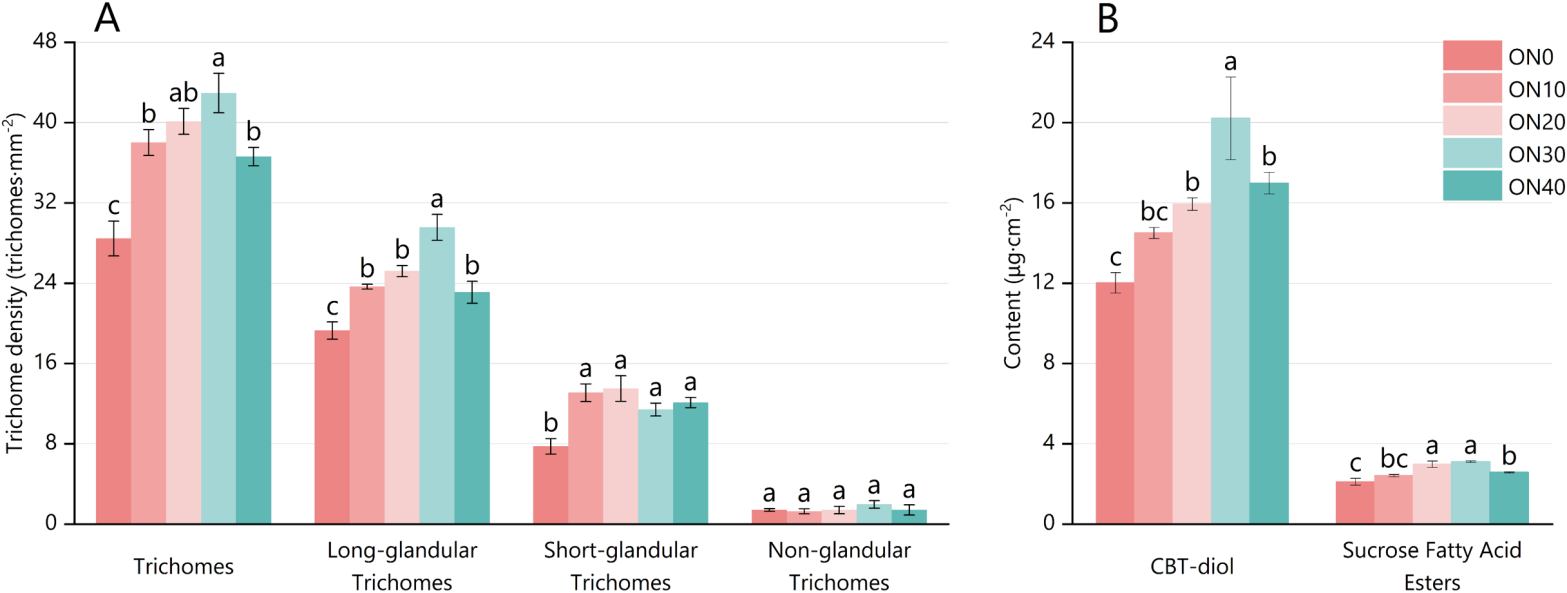
Density of glandular trichomes and content of secretions in tobacco under different treatments. **(A)** Density of total, long-stalked, short-stalked, and non-glandular trichomes. **(B)** Content of cembratriene-diol (CBT-diol) and sucrose fatty acid esters on the leaf surface. The treatments correspond to varying organic nitrogen to total nitrogen ratios: ON0 (0%), ON10 (10%), ON20 (20%), ON30 (30%), and ON40 (40%). Data are presented as the mean ± standard error (SE) of three biological replicates. Different lowercase letters above the bars indicate significant differences among treatments at the p< 0.05 level, as determined by one-way ANOVA followed by Duncan’s multiple range test. CBT-diol: Cembratriene-diol.

During the budding stage, the morphology and density of glandular trichomes on the upper leaves were observed and quantified for each treatment. **Figure 1** shows the density of segmented long-stalked glandular hairs gradually increases from ON0 to ON30, but significantly decreases at ON40. The results showed that glandular trichome density followed the order of ON30 > ON20 > ON10 > ON40 > ON0. Specifically, ON30 exhibited a 50.98% increase in total trichome density compared to ON0 (**Figure 2A**). The density pattern of long-stalked glandular trichomes, key sites for secretion, mirrored that of total trichomes, with ON30 showing a 53.24% increase compared to ON0. Short-stalked trichome density was highest in ON20; while differences among organic nitrogen treatments were not significant, all exceeded ON0 significantly. Non-secretory epidermal trichomes showed no treatment differences.

GC–MS analysis of trichome secretions revealed that the contents of all measured compounds in ON20, ON30, and ON40 were significantly higher than in ON0 (**Figure 2B**). The largest increase occurred in ON30, where cembratriene-diol (CBT-diol) content rose by 68.13% and sucrose esters by 47.39% compared to ON0, paralleling the elevated density of the long-stalked trichomes.

### 3.2 Activities of lipid metabolism-related enzymes

Organic nitrogen ratio significantly influenced lipid-related enzyme activities (**Table 1**). FAS activity peaked in ON30, showing a 27.8% increase over ON0 and exceeded ON10 and ON20 significantly. Although slightly lower in ON40, FAS activity remained significantly above ON0. PAP activity significantly increased only in ON30 (by 44.2% compared to NO0), with other treatments showing non-significant increases. Similarly, PEPC activity also peaked in ON30, rising 82.3% over ON0, and was significantly higher than in all other treatments. Taken together, ON30 (a moderately high level of organic nitrogen) maximized FAS, PAP, and PEPC activities.

**Table 1:** Activities of lipid metabolism-related enzymes in flue-cured tobacco under different organic nitrogen ratios.

Treatment	FAS (nmol·min^-1·^g^-1^)	PAP (μmol·h^-1·^g^-1^)	PEPC (nmol·min^-1^·g^-1^)
ON0	68.24 ± 5.72c	12.31 ± 0.48b	267.24 ± 7.15c
ON10	66.81 ± 0.71c	13.16 ± 0.90b	257.95 ± 1.89c
ON20	72.88 ± 3.76bc	13.40 ± 0.72b	345.12 ± 16.09b
ON30	87.17 ± 4.21a	17.75 ± 0.35a	487.32 ± 1.43a
ON40	81.82 ± 3.73ab	13.80 ± 0.55b	336.55 ± 12.19b

Different lowercase letters following the data in the same column indicate significant differences among treatments at the 0.05 level. The same notation applies to subsequent tables. FAS: Fatty acid synthase, PAP: Phosphatidic acid phosphatase, PEPC: Phosphoenolpyruvate carboxylase.

### 3.3 Content of key lipid metabolism-related products

The contents of PEE, PA, TP, TS, and RS were analyzed in cured leaves (**Table 2**). PEE content was highest in ON30, showing an 18.44% increase compared to Ono, while other treatments did not differ significantly from ON0. PA content decreased significantly in ON30 and ON40 (-17.63% and -5.93%, respectively), whereas ON10 showed a slight increase over ON0. TP content decreased significantly across all treatments compared to ON0, likely reflecting enhanced pyruvate metabolism and downstream diterpenoid biosynthesis, suppressing the phenylpropanoid pathway. Both TS and RS increased with organic nitrogen up to ON30, where they peaked (+43.19% and +64.31% vs. ON0, respectively), before declining in ON40.

**Table 2:** Content of key lipid metabolism-related products in cured tobacco leaves under different organic nitrogen ratios.Units: mg·g^-1^.

Treatment	PEE	PA	TP	TS	RS
ON0	107.37 ± 0.56b	6.24 ± 0.05a	98.54 ± 0.65a	210.66 ± 1.18e	163.86 ± 0.67e
ON10	114.79 ± 1.45b	6.30 ± 0.01a	82.09 ± 0.37c	259.75 ± 2.36d	222.8 ± 1.41d
ON20	114.37 ± 4.03b	6.17 ± 0.09a	84.19 ± 2.33bc	269.66 ± 1.39c	240.37 ± 0.53c
ON30	127.18 ± 1.13a	5.14 ± 0.03c	86.77 ± 0.21b	301.71 ± 0.8a	269.25 ± 0.93a
ON40	112.74 ± 2.16b	5.87 ± 0.04b	84.22 ± 0.56bc	288.24 ± 1.85b	252.49 ± 0.2b

PEE, Petroleum ether extract, PA, Pyruvic acid, TP: Total phenols, TS, Total sugar, RS, Reducing sugar.

### 3.4 Physical properties of cured leaves

Leaf softness, tensile strength, and thickness responded non-linearly to organic nitrogen (**Table 3**). Softness value (inversely related to softness) decreased then increased with rising organic nitrogen, reaching its lowest (softest) point in ON30, significantly below all other treatments. Tensile strength and leaf thickness followed a rising–falling trend, both peaking in ON30. All treatments except ON0 produced leaves within the optimal thickness range (120–140 μm) for upper leaves, with ON30 yielding the thickest leaves.

**Table 3:** Physical properties of cured tobacco leaves under different organic nitrogen ratios.

Treatment	Softness (mN)	Tensile strength (N)	Thickness (μm)
ON0	49.74 ± 0.82a	3.47 ± 0.29c	116.78 ± 2.29d
ON10	41.52 ± 2.31b	3.94 ± 0.15b	125.23 ± 1.93bc
ON20	36.67 ± 0.75c	4.00 ± 0.03b	128.05 ± 0.75b
ON30	30.99 ± 0.97d	4.66 ± 0.07a	136.28 ± 1.52a
ON40	34.62 ± 0.83cd	4.23 ± 0.02ab	121.98 ± 1.52cd

### 3.5 Oil content score

**Figure 3:**
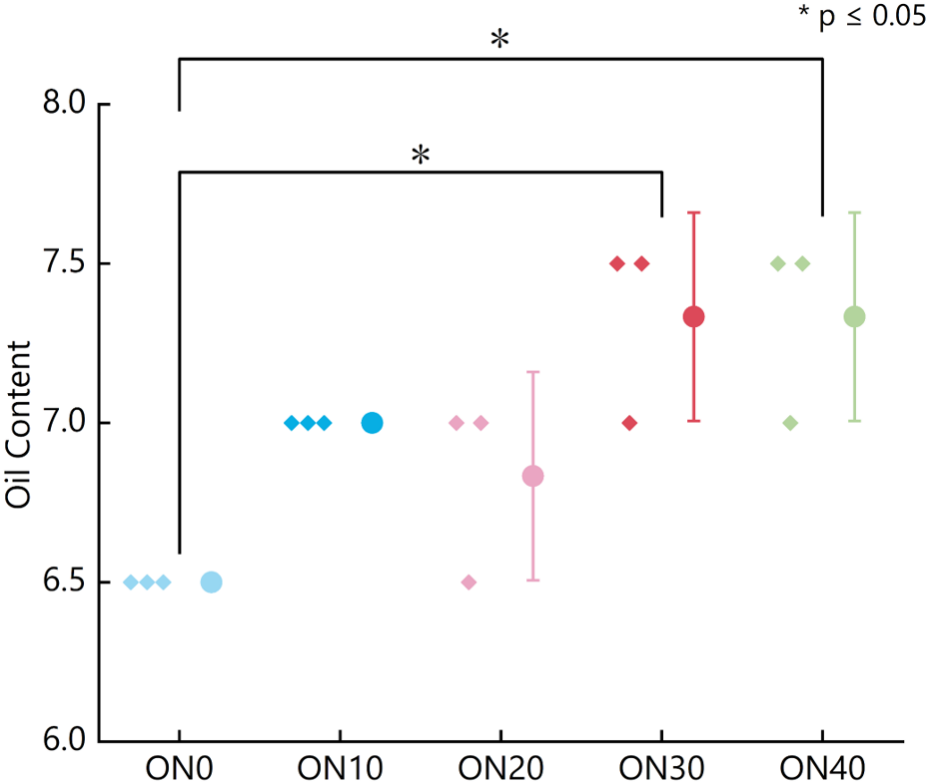
Oil content scores of cured tobacco leaves under different organic nitrogen ratios (original data). The plot displays the oil content scores for each of the five treatments, which correspond to organic nitrogen to total nitrogen ratios of ON0 (0%), ON10 (10%), ON20 (20%), ON30 (30%), and ON40 (40%). Each small point represents an individual replicate score (n=3), while the large colored circle represents the mean score for that treatment. Error bars indicate the standard deviation (SD). An asterisk (*) denotes a statistically significant difference between the indicated groups (p< 0.05), as determined by one-way ANOVA followed by Duncan’s multiple range test.

Oil content scores of cured upper leaves displayed a non-linear response to organic nitrogen (**Figure 3**). ON30 and ON40 achieved the highest scores, both significantly exceeding ON0.

### 3.6 Correlation analysis

**Figure 4:**
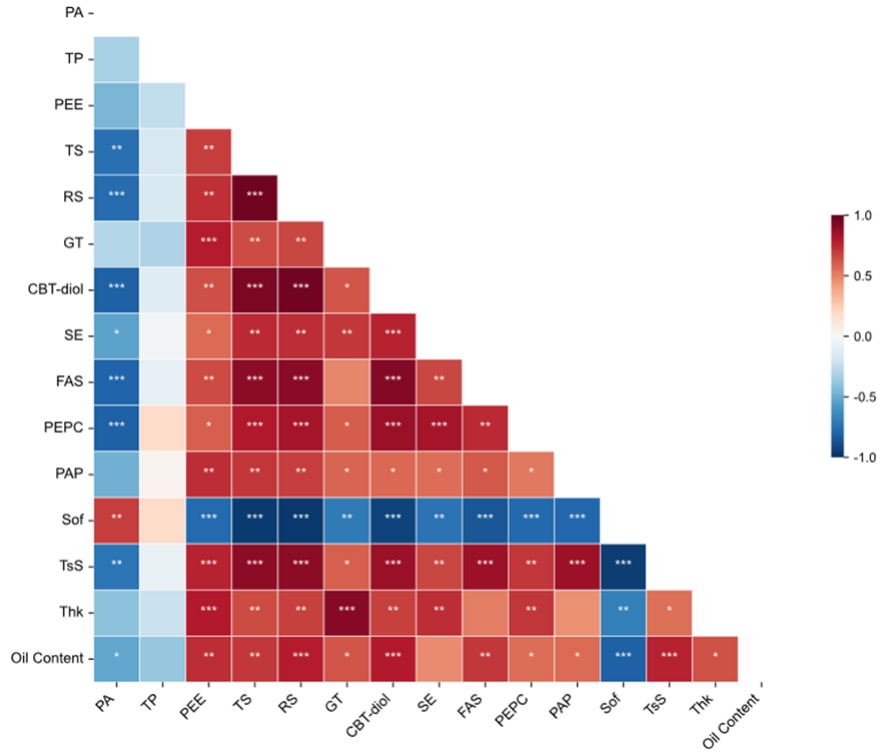
Spearman correlation analysis of physicochemical indicators and oil content. The heatmap displays the pairwise Spearman’s rank correlation coefficients. The color gradient represents the correlation coefficient value, with red indicating a positive correlation and blue indicating a negative correlation. Asterisks denote statistical significance (*p< 0.05, **p< 0.01, ***p< 0.001). PA: Pyruvic acid, TP: Total phenols, PEE: Petroleum ether extract, TS: Total sugar, RS: Reducing sugar, GT: Glandular trichomes density, CBT-diol: Cembratriene-diol, SE: Sucrose esters, FAS: Fatty acid synthase, PEPC: Phosphoenolpyruvate carboxylase, PAP: Phosphatidic acid phosphatase, Sof: Softness, TsS: Tensile strength, Thk: Thickness.

Spearman correlation analysis (**Figure 4**) was conducted to explore the relationships among oil content and physicochemical indicators. As shown in the heatmap, oil content exhibited significant positive correlations (p< 0.05) with PEE, FAS, TS, RS, Glandular trichomes density (GT), CBT-diol, PEPC, PAP, and tensile strength (TsS). Conversely, it showed significant negative correlations with PA content and softness value (Sof). Notably, strong multicollinearity was observed between certain features, such as TS and RS, necessitating feature selection for subsequent modeling.

### 3.7 Random forest model performance and diagnostics

To construct a robust predictive model despite the limited sample size, we employed a data augmentation strategy combined with rigorous feature selection, and encoded the treatments as 0, 10, 20, 30, 40.

**Figure 5:**
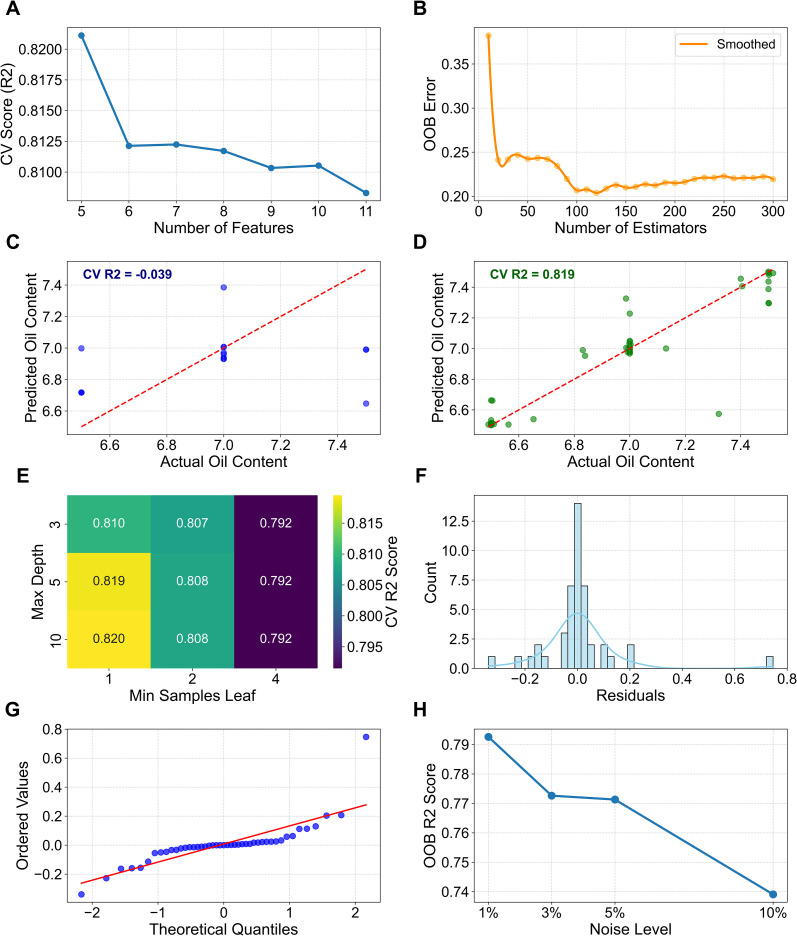
Performance and diagnostic plots for the random forest model. All analyses were performed on the augmented dataset. **(A)** RFE Feature Selection Performance, illustrating the cross-validation score (R²) as a function of the number of selected features. **(B)** Out-of-Bag (OOB) error curve, showing the smoothed model error rate as the number of estimators (trees) increases. **(C)** Cross-validation predicted versus actual oil content values using the original dataset (N = 15), illustrating the model’s inability to generalize due to limited sample size (CV R^2^=−0.039). **(D)** Cross-validation predicted versus actual values using the augmented dataset (N = 45), demonstrating significantly improved predictive accuracy and the effectiveness of the augmentation strategy (CV R^2^ = 0.819). **(E)** Grid Search Performance (CV R²), displaying the impact of max_depth and min_samples_leaf on model accuracy. **(F)** Residuals Distribution, showing the frequency of residual values with a fitted normal distribution curve. **(G)** Quantile-Quantile (Q-Q) plot of the CV residuals against a theoretical normal distribution. **(H)** Sensitivity of Model Performance to Data Augmentation Noise, demonstrating the stability of OOB R² across different noise levels.

Recursive Feature Elimination (RFE) with cross-validation was first applied to the augmented dataset. As shown in **Figures 5A, the** model’s performance stabilized when the number of features was reduced to five. Consequently, PEE, CBT-diol, Sof, RS, and GT were selected as the optimal input features for the final model, effectively reducing redundancy while retaining critical biological information.

The Random Forest model trained on this optimized feature set and augmented data demonstrated excellent performance. Grid search optimization (**Figure 5E**) identified the optimal hyperparameters (e.g., n_estimators=300, max_depth=10, min_samples_leaf=1), which were used for the final training. The model achieved an OOB R² of 0.781 and a 3-fold CV R² of 0.819. In stark contrast, the model trained on the original small dataset (N = 15) failed to generalize, yielding a negative CV R² (-0.039) (**Figures 5C, D**). This comparison underscores the necessity and effectiveness of our data augmentation strategy. The OOB error curve (**Figure 5B**) further confirmed that the error rate stabilized and minimized around 150–200 trees, validating the sufficiency of setting n_estimators to 300.

Model diagnostics indicated that the assumptions for regression were met. The distribution of residuals was approximately normal (**Figure 5F**), and the Q-Q plot (**Figure 5G**) showed that the standardized residuals aligned well with the theoretical quantiles, with no significant deviations. Sensitivity analysis (**Figure 5H**) demonstrated that the model’s performance remained relatively stable across different noise levels (1% - 3%) used in augmentation, although performance degraded at very high noise levels (10%), confirming the appropriateness of the chosen 3% noise parameter.

### 3.8 Identification of key physicochemical indicators

**Figure 6:**
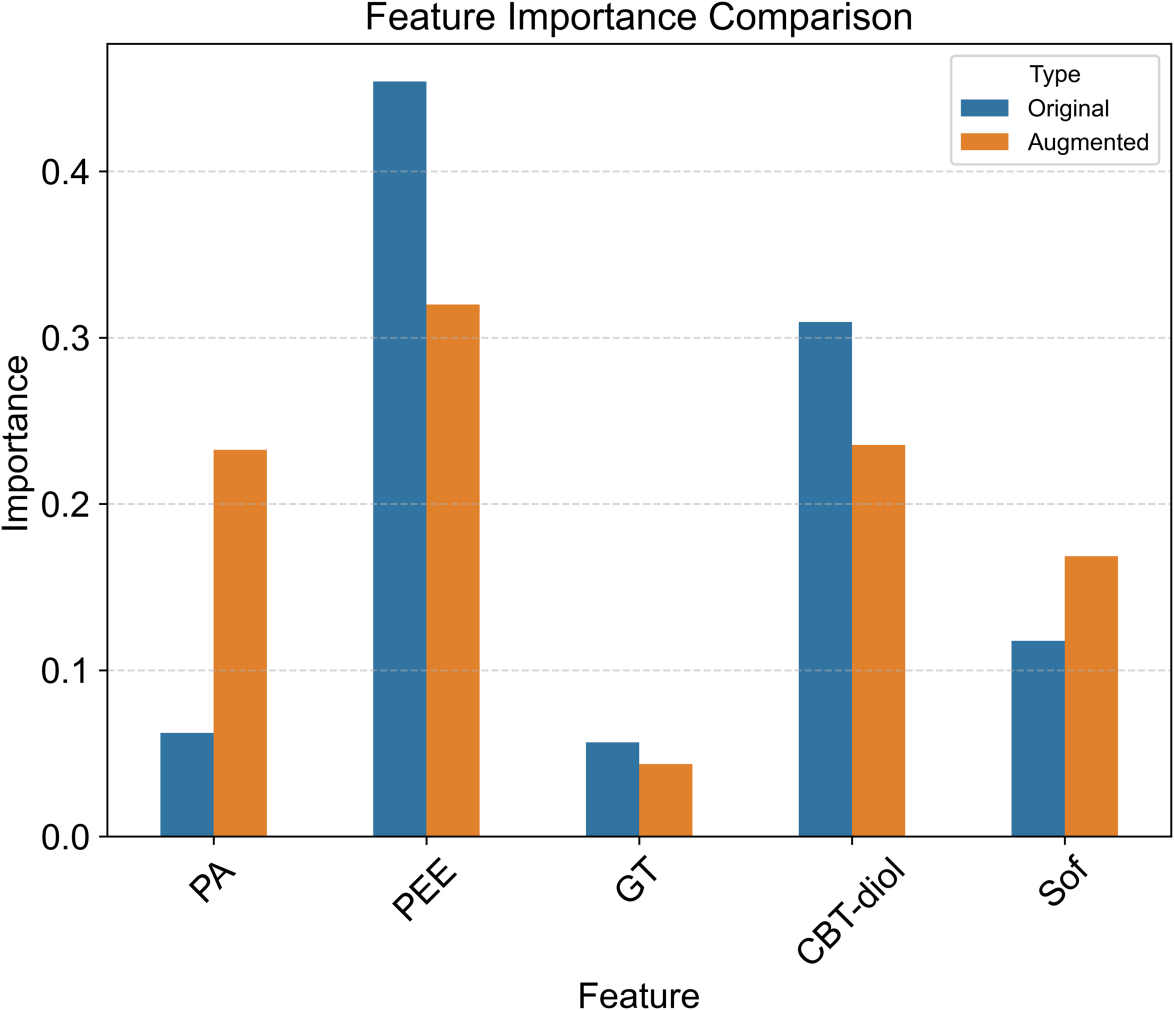
Feature importance comparison. This bar chart compares the importance of features derived from the Random Forest model trained on the original dataset versus the augmented dataset. The importance is measured by the Mean Decrease in Impurity (MDI). PA: Pyruvic acid, PEE: Petroleum ether extract, GT: Glandular trichomes density, CBT-diol: Cembratriene-diol, Sof: Softness.

Feature importance analysis based on Mean Decrease in Impurity (MDI) (**Figure 6**) revealed the relative contribution of each indicator to the oil content prediction. PEE was identified as the most influential predictor, followed closely by CBT-diol and Sof. The feature importance rankings between the trained models based on the raw data and enhanced data are almost consistent, further verifying the robustness of identifying key features.

### 3.9 Mechanistic interpretation of key indicators’ effects on tobacco oil content

To gain a deeper understanding of how key indicators influence oil content, we interpreted the RF model using Partial Dependence Plots (PDP) and SHAP value analysis.

**Figure 7:**
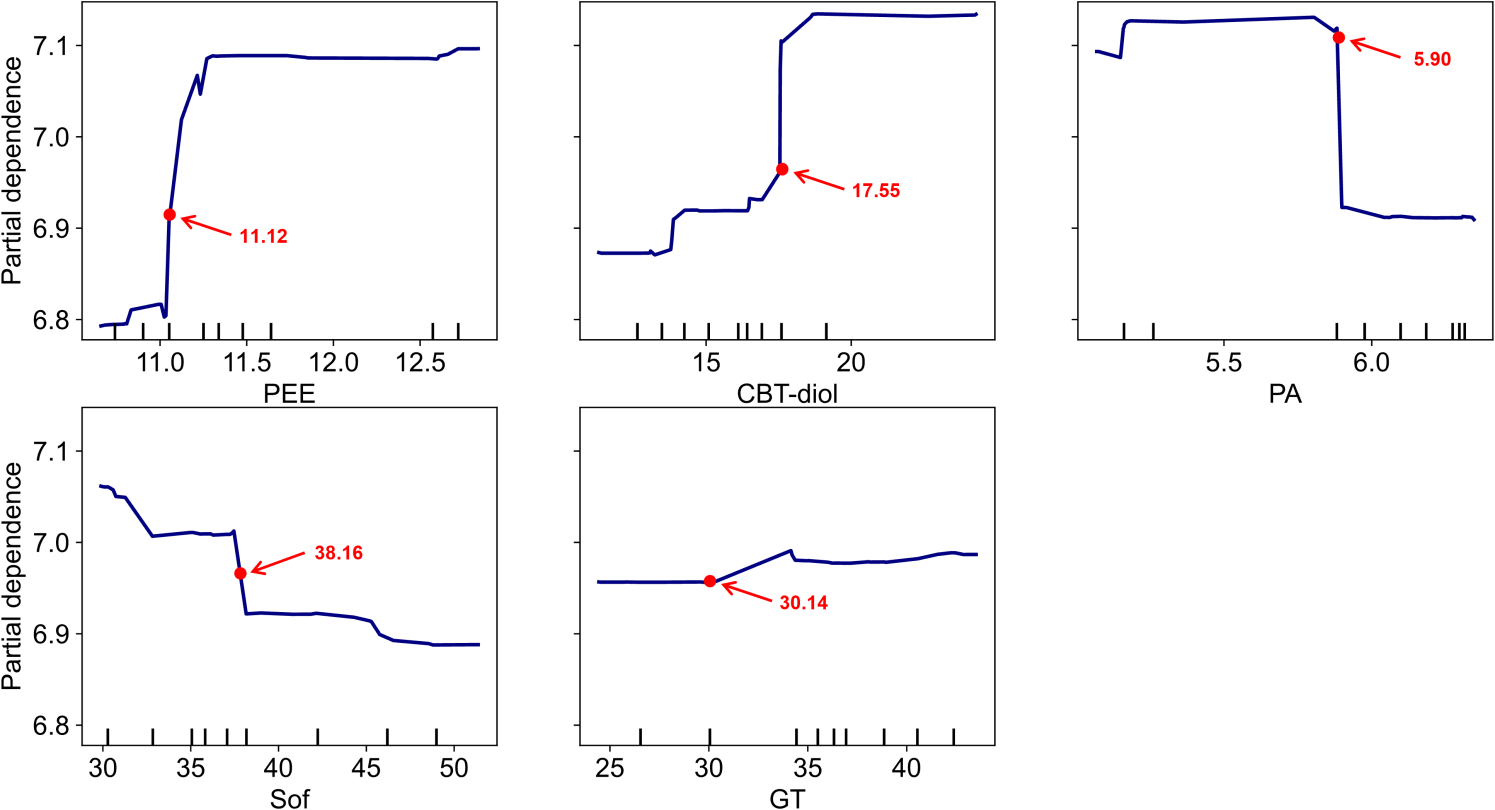
Partial dependence plots (PDP) of key indicators on predicted oil content with inflection points. The panels display the marginal effects of the top five features on the predicted oil content score, as determined by the Random Forest model trained on the augmented dataset. The y-axis represents the average partial dependence, and the x-axis corresponds to the value of each feature on its original scale. Red dots indicate the primary inflection points (points of maximum gradient change), with their specific values annotated, highlighting critical thresholds where feature influence shifts most dramatically. Rug plots at the bottom show the distribution of the augmented data points. PEE: Petroleum ether extract, CBT-diol: Cembratriene-diol, PA: Pyruvic acid, Sof: Softness, GT: Glandular trichomes density.

The PDP (**Figure 7**) revealed the marginal effects of the top five features. PEE and CBT-diol demonstrated strong positive associations with oil content: as their values increased, the predicted oil content score rose sharply before leveling off, suggesting a saturation effect. GT also showed a positive trend. In contrast, Sof exhibited a clear negative relationship, where lower softness values (indicating softer leaves) were associated with higher oil scores. PA showed a threshold effect, where oil scores dropped significantly when PA content exceeded a certain level.

**Figure 8:**
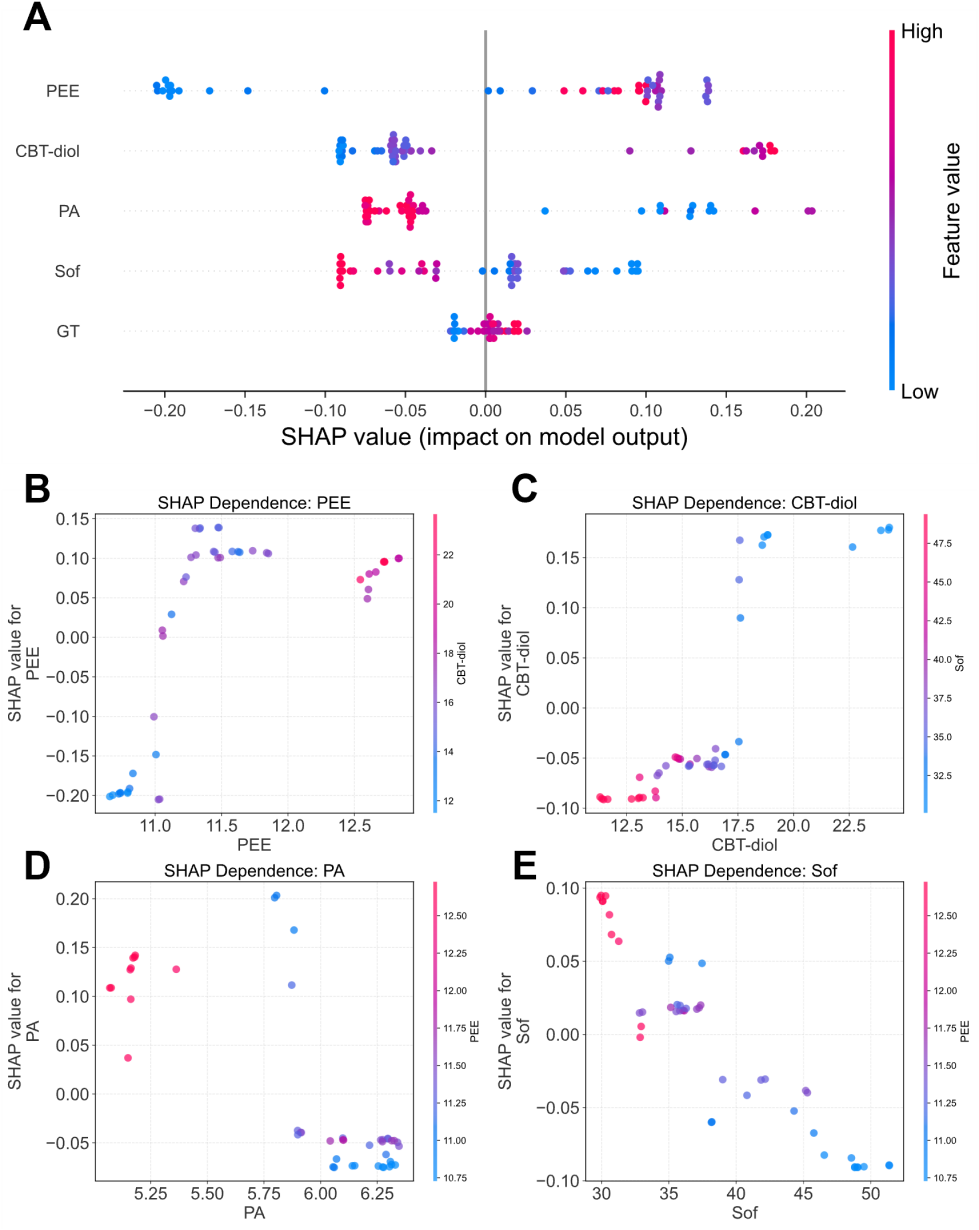
SHAP value analysis of the random forest model. **(A)** SHAP Summary Plot. Each point represents a single sample. The position on the y-axis indicates the feature, and the position on the x-axis indicates the SHAP value (impact on model output). The color represents the feature value (red for high, blue for low). **(B-E)** SHAP Dependence Plots Illustrating Interaction Effects. Each panel plots the SHAP value of a primary feature against its own value. The color of the points is determined by the value of a second, interacting feature. **(B)** SHAP dependence for PEE colored by CBT-diol. **(C)** SHAP dependence for CBT-diol colored by Sof. **(D)** SHAP dependence for PA colored by PEE. **(E)** SHAP dependence for Sof colored by PEE. PEE: Petroleum ether extract, CBT-diol: Cembratriene-diol, PA: Pyruvic acid, Sof: Softness, GT: Glandular trichomes density.

SHAP analysis provided further insights into individual feature contributions and interactions. The SHAP summary plot (**Figure 8A**) confirmed the global importance trends, showing that high values of PEE, CBT-diol, and GT had a positive impact on the model output (pushing it to the right), while high values of Sof and PA had a negative impact. We further explored the interactions among the top four features using SHAP dependence plots (**Figures 8B–E**), which revealed that the influence of individual indicators is highly context-dependent. For instance, the positive contribution of PEE to oil content was notably modulated by CBT-diol, specifically, samples characterized by both high PEE and high CBT-diol levels exhibited elevated SHAP values, suggesting a synergistic effect between these lipid-related metabolites. A similar but negative, more complex, interaction was observed between CBT-diol and leaf softness. Differently, the negative effects of Sof and PA on oil content were found to vary depending on the levels of PEE and Sof, respectively. These results underscore that tobacco oil content is not determined by single factors in isolation, but rather by the complex interplay among lipid metabolites, physical properties, and precursor availability.

### 3.10 Data structure and model

**Figure 9:**
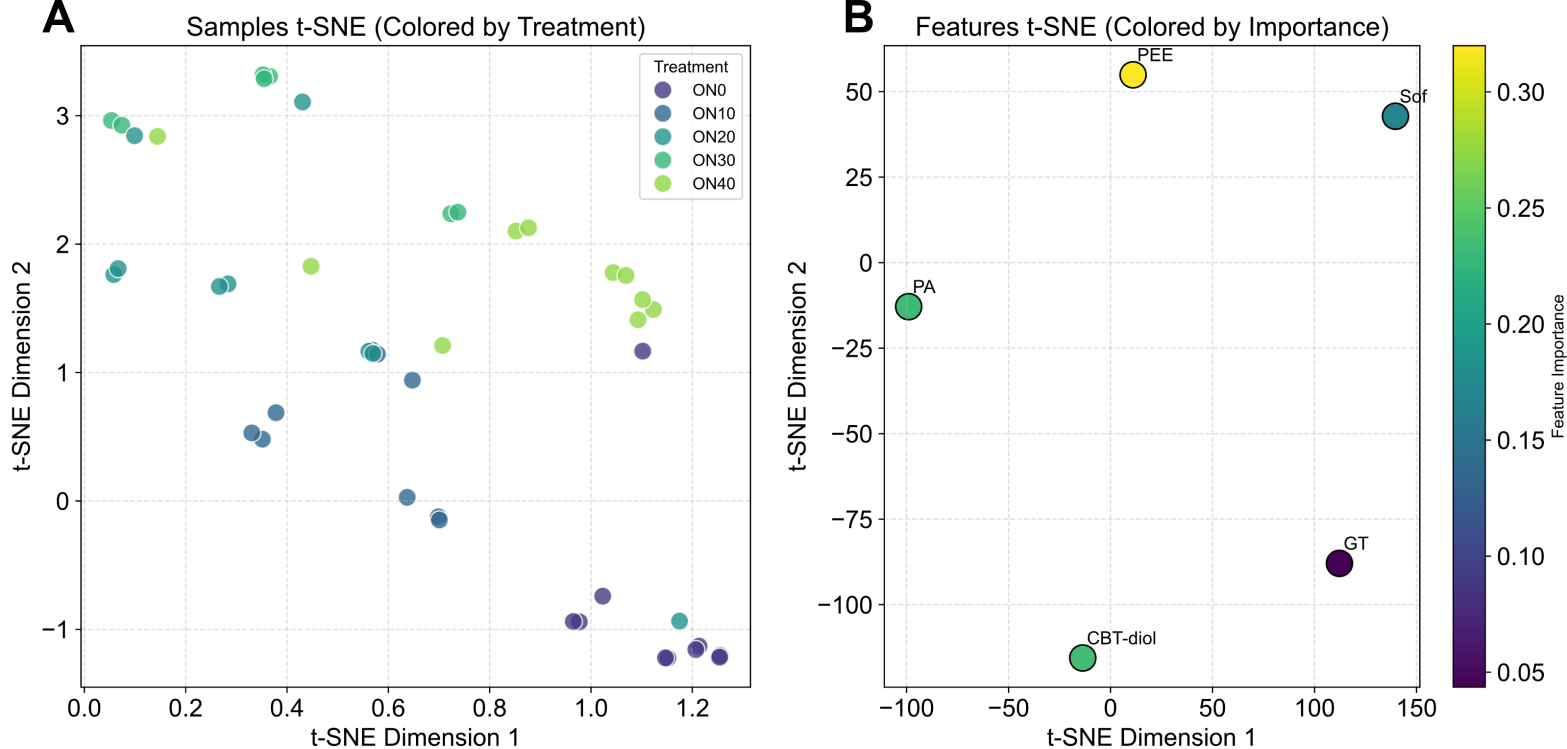
t-SNE dimensionality reduction visualization of augmented data. **(A)** t-SNE plot of the augmented samples (N = 45), colored by their respective organic nitrogen treatment level (% Org. N). This visualization confirms that the data augmentation strategy preserved the distinct clustering patterns of different treatments in the high-dimensional feature space. **(B)** t-SNE plot of the physicochemical features, colored by their feature importance score from the Random Forest model. Proximity between features may suggest similar functional roles or response patterns. PA, Pyruvic acid; PEE, Petroleum ether extract; GT, Glandular trichomes density; CBT-diol, Cembratriene-diol; Sof, Softness.

t-SNE visualization (**Figure 9**) was employed to explore the underlying data structure. **Figure 9A** shows that samples from different organic nitrogen treatments (ON0-ON40) formed distinct clusters or gradients in the 2D space, confirming that the selected five features contain sufficient information to distinguish between treatments. **Figure 9B** displays the features in the t-SNE space, colored by their importance. The proximity of certain features (e.g., GT and CBT-diol) suggests they may share similar information or biological roles in determining leaf quality.

**Figure 10:**
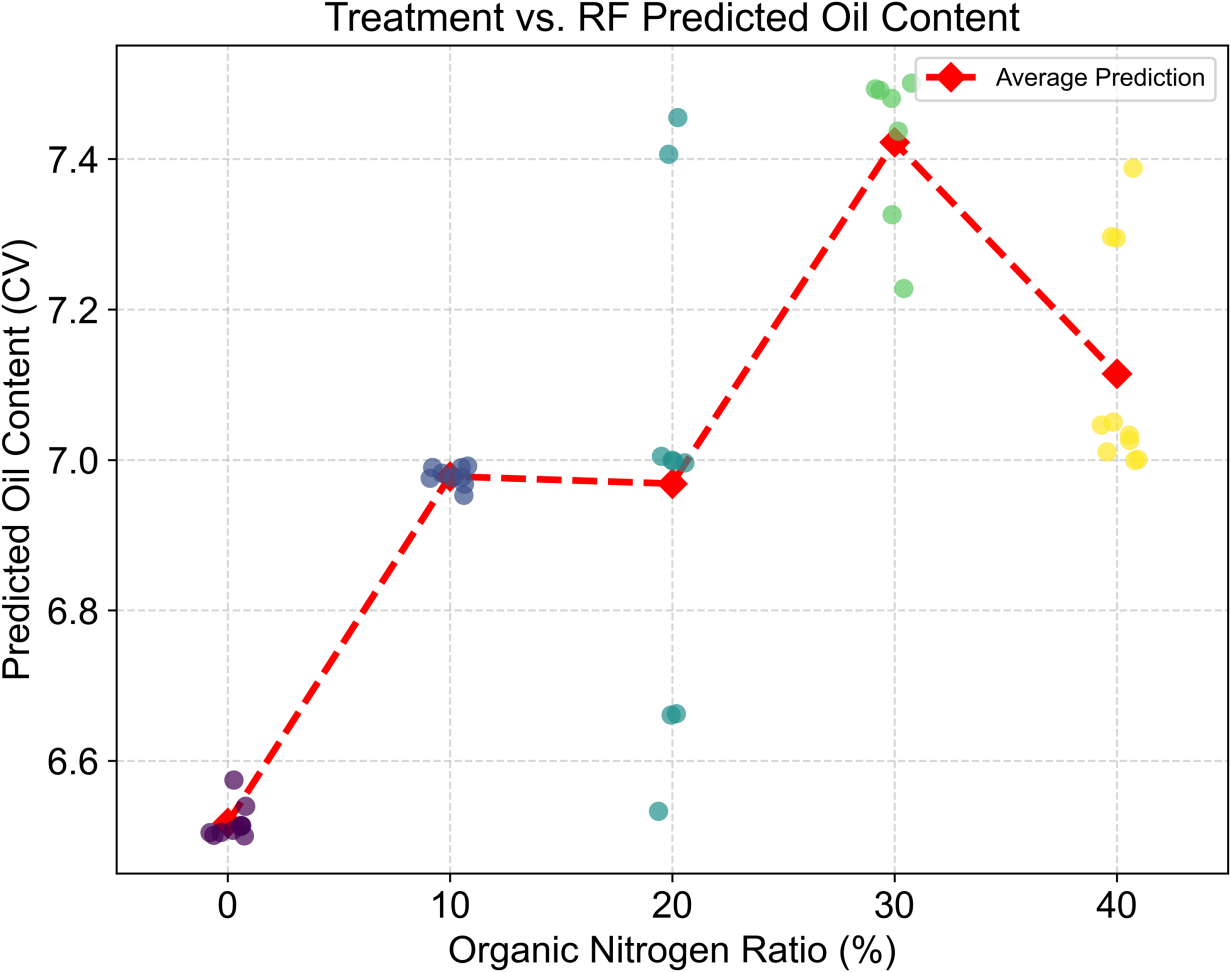
Relationship between treatment level and cross-validated predictions of oil content (augmented data). The plot displays the cross-validation (CV) predicted oil content scores from the Random Forest model for each sample in the augmented dataset (N = 45) under different organic nitrogen ratios. Each point represents a single augmented sample, with its color indicating the predicted oil content value. The dashed red line connects the average predicted oil content for each treatment level, illustrating the overall trend captured by the model.

Finally, we compared the treatment levels with the RF model’s cross-validation predictions (**Figure 10**). The average predicted oil content for each treatment level accurately reproduced the non-linear trend observed in the actual field experiment: rising from ON0 to a peak at ON30, and then slightly declining at ON40. This confirms that our machine learning model successfully captured the true biological response of tobacco oil content to varying organic nitrogen application ratios.

## 4 Discussion

### 4.1 Glandular trichomes and secretions as primary drivers of oil content

The secretory trichomes (glandular trichomes) on the leaf surface of flue-cured tobacco at specific developmental stages are a key factor influencing oil content and overall quality. Glandular trichomes are the sites for the synthesis and secretion of numerous very-long-chain fatty acids, their derivatives, and alicyclic compounds (Philippe et al., 2022; Wang, 2014). Among these, cembratriene-diol and sucrose esters are important secretions of the glandular trichomes. Both are crucial aroma precursors that can impart desirable aroma and sweetness to flue-cured tobacco (Qu et al., 2018), and they enhance the “oily” sensation of the leaf surface, contributing to the improvement of the tobacco’s appearance quality. As the primary secretory organs for terpenoids, an increased density of long-stalked glandular trichomes directly thickens the waxy layer on the leaf surface, thereby enhancing the tactile perception of oiliness (e.g., smoothness) and the aroma (Wang et al., 2022). In this study, all treatments with increased organic nitrogen ratios showed significantly greater densities of all types of glandular trichomes and higher secretion contents compared to the control. Notably, in ON30, the density of long-stalked glandular trichomes increased by 53.24% compared to ON0, with a concurrent significant rise in the contents of CBT-diol and sucrose ester secretions. This is likely the reason why the final oil content score for ON30 was significantly higher than that for ON0. Our Random Forest model further validated this biological observation: Recursive Feature Elimination (RFE) identified both Glandular Trichome density (GT) and CBT-diol content as top-tier predictors (**Figure 5A**), confirming their indispensable role in determining the final oil score. Some researchers suggest that the content of glandular trichome secretions may be related to the regulation of cell wall structure by the combined application of organic nitrogen (Zhou et al., 2024). For instance, the increased leaf thickness observed in ON30 might enhance the mechanical strength of the leaf, reducing physical damage to the trichomes during growth and thus allowing for the retention of more secretions. However, this mechanism requires further verification.

### 4.2 Lipid metabolism as a core biochemical pathway

Carbohydrate-derived pyruvic acid (PA) serves as a centralxnode linking glycolysis to lipid biosynthesis, acting as a precursor for acetyl-CoA, fatty acid chains, sterols, and terpenes via the tricarboxylic acid (TCA) cycle and methylerythritol phosphate (MEP) pathway (Li-Beisson et al., 2010). The synthesis of TP, secondary metabolites derived from PA metabolism, competes with lipid synthesis for common precursors like phosphoenolpyruvate (PEP). In ON30, PA levels declined sharply (−17.6%), suggesting enhanced carbon flux toward acetyl-CoA and downstream lipid synthesis (**Table 2**). This reduction is consistent with the hypothesis that carbon flux may be reallocated from the PA pool towards acetyl-CoA via the TCA cycle for lipid synthesis or towards other downstream products (Chen and Zhu, 2023), although this metabolic flux shift is inferred from static concentration changes. The enhancement of this metabolic pathway might indirectly lead to the downregulation of the phenylpropanoid pathway, thus explaining the decrease in TP content. PEPC is a key enzyme in carbon fixation and the supply of precursors for amino acid and organic acid synthesis, influencing overall metabolic flux and ultimately affecting the production of secondary metabolites (Baena et al., 2021). An increase in its activity not only accelerates the conversion of PA but also promotes the accumulation of TS and RS by enhancing carbon-nitrogen synergy. FAS plays a central role in the *de novo* synthesis of fatty acids, and its increased activity directly promotes fatty acid accumulation (Kitajima-Koga et al., 2020). Meanwhile, PAP is involved in lipid metabolism and the regulation of phospholipid levels, and the reactions it participates in are crucial steps in the synthesis and storage of triacylglycerols (Torres-Rodríguez et al., 2021). This study found that ON30 significantly enhanced the activities of FAS, PAP, and PEPC, which is likely a key factor contributing to the significant increase in the oil content score for this treatment. The significant increase in PEE content in ON30 and its strong correlation with oil content further corroborate this conclusion, as PEE comprises nearly all secondary metabolites in tobacco leaves (Jiang et al., 2025) and is often used as a proxy indicator for the “oil content” of flue-cured tobacco. However, a notable decline in enzyme activities (FAS, PEPC) and lipid metabolites (PEE, CBT-diol) was observed at the highest organic nitrogen level (ON40). This “diminishing return” or inhibitory effect likely stems from the carbon-nitrogen imbalance hypothesis. Excessive organic nitrogen input in ON40 may lead to an overabundance of nitrogen relative to carbon skeletons. According to the C/N balance theory, plants prioritize carbon resources for amino acid and protein synthesis to assimilate excess nitrogen, thereby diverting acetyl-CoA away from the lipid biosynthesis pathway (Fritz et al., 2006). Additionally, the significantly lower soil pH observed under high organic loading (ON40) might create a sub-optimal rhizosphere environment, suppressing the activity of soil microorganisms and root enzymes essential for nutrient uptake and metabolism (Feng et al., 2023). Meanwhile, it is crucial to acknowledge a potential spatial mismatch: while our enzyme activity assays represent whole-leaf protein levels, lipid biosynthesis relevant to “oiliness” is highly concentrated in the glandular trichomes. Future studies utilizing trichome-specific metabolite profiling are needed to directly link enzyme expression with localized lipid accumulation.

### 4.3 Synergistic effects of leaf physical properties

The sensory evaluation of oil content depends not only on chemical composition but is also closely related to the physical properties of the tobacco leaf (Cai et al., 2023). In our study, ON30 significantly reduced the softness value (**Table 3**), indicating that the leaves from this treatment were softer, which is consistent with the typical tactile characteristics of high-oil-content leaves (Li et al., 2008). The increase in tensile strength reflects an optimization of the leaf’s fibrous structure, which may be attributed to the regulation of cell wall components (such as cellulose and pectin) by the combined application of organic nitrogen (Ogden et al., 2018). Furthermore, the significant increase in leaf thickness for ON30, while remaining within the standard range (120-140 μm), ensured both good combustion performance and avoided difficulties in rolling due to excessive thickness. These physical traits contribute directly to the perception of oiliness, highlighting the multifactorial nature of oil content, where chemical and structural factors act synergistically. The deterioration of these physical traits (increased hardness, decreased thickness) in ON40 further supports the notion of a physiological stress response to nutrient over-supply, where rapid vegetative growth might occur at the expense of structural refinement and secondary metabolite accumulation. Importantly, our RF model identified Softness (Sof) as a top-three predictor. While softness itself is a physical state rather than a metabolic driver like FAS, it serves as a critical “accompanying trait” that integrates the outcomes of cell wall structure and moisture retention, making it a reliable indicator for sensory quality.

### 4.4 Insights from random forest model

Despite the inherent challenges of small sample sizes in agricultural field trials, the Random Forest (RF) model, augmented with a rigorous data generation strategy (noise injection and linear interpolation), demonstrated robust explanatory power. The stark contrast between the poor performance on original data (CV R² = -0.039) and the high accuracy on augmented data (CV R² = 0.819) highlights the necessity of data augmentation in capturing latent biological patterns that are otherwise obscured by limited sampling (Shorten and Khoshgoftaar, 2019). By integrating RFE for feature selection, we distilled the model down to five core indicators—PEE, CBT-diol, Sof, PA, and GT—which collectively explain the majority of the variance in oil content.

The feature importance analysis (**Figure 6**) and PDP (**Figure 7**) quantified the relative contributions of these markers. PEE and CBT-diol emerged as the most dominant positive drivers, confirming that the abundance of lipid-related metabolites is the material basis of oiliness. Interestingly, PA exhibited a threshold-dependent negative effect, consistent with its role as a transient metabolic precursor that is depleted during active lipid synthesis (Zeng et al., 2025).

Crucially, the SHAP interaction analysis (**Figure 8**) moved beyond single-factor effects to reveal context-dependent regulation. We observed that the positive contribution of PEE to oil content was synergistic with CBT-diol levels. This reflects the physiological reality where PEE (a solvent mixture) and CBT-diol (a specific solute) co-vary to determine the sensory “oiliness.” Similarly, the negative impact of Softness appeared to be modulated by PEE levels, suggesting that high lipid content might partially compensate for less-than-ideal leaf texture. These non-linear and interactive insights provide a more nuanced understanding of quality formation than traditional linear correlation analyses.

### 4.5 Study limitations and future directions

While this study offers valuable insights into the regulation of tobacco oil content, several limitations must be acknowledged. First, the field experiment was conducted at a single location over one growing season. Given the known influence of environmental factors (temperature, rainfall, soil type) on secondary metabolism, multi-year and multi-location trials are necessary to validate the generalizability of the optimal 30% organic nitrogen ratio. Second, although the data augmentation strategy successfully mitigated overfitting and improved model convergence (as evidenced by the OOB error curve), the original sample size (N = 15 biological replicates) remains small for machine learning applications. The findings should be interpreted as exploratory hypothesis generation rather than definitive predictive rules. Future research should prioritize larger-scale datasets and incorporate multi-omics approaches (transcriptomics and metabolomics) to construct a comprehensive regulatory network spanning genes, metabolites, and sensory traits.

## 5 Conclusion

In conclusion, this study demonstrates that optimizing the organic nitrogen ratio, particularly at 30%, enhances flue-cured tobacco oil content via coordinated effects on glandular trichome development, lipid metabolism, and leaf physical properties. By integrating field experiments with a Random Forest model trained on augmented data, we successfully captured the non-linear, interactive pathways underpinning oil content regulation. PEE, CBT-diol, and Softness emerged as the most robust indicators, with PEE and CBT-diol acting as primary material drivers and Softness serving as a key accompanying physical trait. These findings establish a data-driven framework for improving tobacco leaf quality and provide a theoretical basis for precise nitrogen management. Future work should focus on validating these mechanisms across diverse production environments and employing gland-specific metabolic profiling.

## Data Availability

The original contributions presented in the study are included in the article/**Supplementary Material**. Further inquiries can be directed to the corresponding authors.
